# Complexes of Cu(II) Ions and Noncovalent Interactions in Systems with L-Aspartic Acid and Cytidine-5'-Monophosphate

**DOI:** 10.1155/2008/253971

**Published:** 2008-07-30

**Authors:** Romualda Bregier-Jarzebowska, Anna Gasowska, Lechosław Lomozik

**Affiliations:** Faculty of Chemistry, Adam Mickiewicz University, Grunwaldzka 6, 60-780 Poznań, Poland

## Abstract

Interactions between aspartic acid (Asp) and cytidine-5-monophosphate (CMP) in metal-free systems as well as the coordination of Cu(II) ions with the above ligands were studied. The composition and overall stability constants of the species formed in those systems were determined by the potentiometric method, and the interaction centres in the ligands were identified by the spectral methods UV-Vis, EPR, NMR, and IR. In metal-free systems, the formation of adducts, in which each ligand has both positive and negative reaction centres, was established. The main reaction centres in Asp are the oxygen atoms of carboxyl groups and the nitrogen atom of the amine group, while the main reaction centre in CMP at low pH is the N(3) atom. With increasing pH, the efficiency of the phosphate group of the nucleotide in the interactions significantly increases, and the efficiency of carboxyl groups in Asp decreases. The noncovalent reaction centres in the ligands are simultaneously the potential sites of metal-ion coordination. The mode of coordination in the complexes formed in the ternary systems was established. The sites of coordination depend clearly on the solution pH. In the molecular complexes ML⋯L, metallation involves the oxygen atoms of the carboxyl groups of the amino acid, while the protonated nucleotide is in the outer coordination sphere and interacts noncovalently with the anchoring CuH_*x*_(Asp) species. The influence of the metal ions on the weak interactions between the biomolecules was established.

## 1. INTRODUCTION

Interactions between metal ions and nucleic acids or their fragments affect the character of many biological processes including that of genetic information transfer [1–6]. The effective centres of coordination with metal ions are the donor nitrogen atoms N(3) from pyrimidine bases and the oxygen atoms from the phosphate groups of the nucleotide. These centres are also the sites of noncovalent interactions with the other bioligands present in living organisms, such as small organic polycations, polyamines, or amino acids [7, 8].

Aspartic acid (Asp) is a naturally occurring amino acid. Along with glutamic acid, it acts as a neurotransmitter in the central nervous system [9–14]. Aspartic acid takes part in thermogenic processes induced by prostaglandin E_1_ (PGE_1_) [15] and is a component of the active centres of some enzymes. It influences the solubility and ionic character of proteins, protects the liver against the toxic effect of drugs, participates in the generation of ribonucleotides thereby enhancing the effectiveness of the immunological system of the organism, and prevents the destruction of neurons and the brain. The presence of metal ions in living organisms modifies the character of bioprocesses. The reactions between the amino acid and the metal ions are considered as models of the processes which take place at the molecular level in the metal/protein system.

Although studies of the systems of metals with dicarboxylic amino acids have been carried out since the 1970s, [16–19], no definite conclusions as to the mode of coordination have been obtained, in particular in the ternary systems, which is related to the fact that aspartic acid has three functional groups (one amine group and two carboxyl ones). To our best knowledge, no information has been reported on interactions in the metal-free systems of aspartic acid/nucleotide or on the character of interactions in ternary systems including metal ions.

This paper presents the results of a study on the coordination of Cu(II) ions with aspartic acid and cytidine-5′-monophosphate (CMP) and the interactions of these bioligands in metal-free systems.

## 2. EXPERIMENTAL

Cytidine 5′-monophosphate, C_9_H_14_N_3_O_8_P, and L-aspartic acid, C_4_H_7_NO_4_, were purchased from Sigma-Aldrich and were used without further purification. Cu(NO_3_)_2_ bought in POCH Gliwice (Poland) was twice recrystallized from H_2_O before use. The method of determination of Cu(II) concentration in a parent solution of a concentration of about 2.4 × 10^−2^ M was described earlier [20, 21]. Potentiometric studies were performed on a Methrom 702 SM Titrino with a glass electrode Methrom 6.0233.100 calibrated in terms of hydrogen ion concentration [22] with a preliminary use of borax (pH = 9.225) and phthalate (pH = 4.002) standard buffers. The concentrations of the CMP and Asp were 5·10^−3^ in the metal-free systems and from 1·10^−3^ to 2.5·10^−3^ M in the systems with Cu(II). The ratio of ligand1:ligand2 in the metal-free systems was 1:1, metal:ligand1 was 1:2.5, and metal:ligand1:ligand2 were 1:1:1 or 1:2.5:2.5 in the ternary systems (ligand1-Asp,ligand2-CMP). Potentiometric titrations were performed at the ionic strength *μ* = 0.1 M (KNO_3_), at 20 ± 1°C under helium, using as a titrant CO_2_-free NaOH solution (about 0.2 M). For each system a series of 10 titrations was made; the initial volume of the sample was 30 cm^3^. No precipitate formation was, observed in the entire pH range studied. Calculations were performed using 100–350 points for each job. The selection of the models and the determination of the stability constants of the complexes were made using the SUPERQUAD program [23], whereas the distribution of particular forms was determined by the HALTAFALL program [24]. The computer procedures used for the purpose, choice of the models. and the criteria of verification of results are described in [25–29]. The samples for ^13^C NMR and ^31^P NMR investigation were prepared by dissolving appropriate amounts of ligands and Cu(NO_3_)_2_ in D_2_O and adjusting pH by the addition of NaOD (or (C_2_H_5_)_4_NOH) and DNO_3_, correcting pH-readings (a pH-meter C5-501 made by Elmetron) according to the formula: pD = pH_readings_ + 0.40 [30]. The concentration of the ligands in the samples was 0.05 M, and the concentration ratio of Cu(II) to CMP and Asp was 1:200:200. ^13^C NMR spectra were recorded on an NMR Gemini 300 VT Varian spectrometer using dioxane as an internal standard. The positions of ^13^C NMR signals were converted to the TMS scale. ^31^P NMR spectra were taken on an NMR Varian Unity 300 spectrometer with H_3_PO_4_ as a standard. UV-Vis spectra were taken on a UV 160 Shimadzu spectrometer for the ligand and metal concentrations of the same value as in the samples for potentiometric titrations. Electron spin resonance (EPR) spectra were taken at 77 K in a water-glycol solution (3:1, v/v) on a Radiopan SE/X 2547 spectrometer (C_Cu^2+^_ = 0.002 M) at the ratio of metal:amino acid ratio 1:4 and metal:nucleotide:amino acid ratio 1:2.5:2.5. IR measurements were carried out using a Bruker ISS 66vS spectrophotometer.

The hydrolysis constants for the metal ion Cu(II) were taken from [31] and were fully employed in the calculations.

**Scheme 1:**
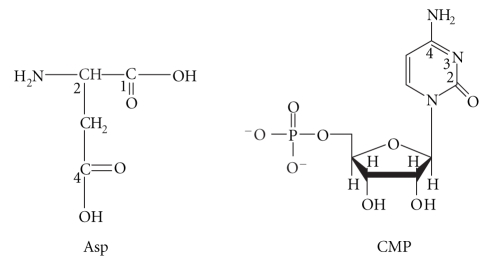
Chemical formulae of the bioligands studied.

The ligands studied are presented in Scheme 1.

## 3. RESULTS AND DISCUSSION

In the systems of polyamine/nucleotide studied earlier at our laboratory, noncovalent interactions were observed in the pH ranges in which one ligand was deprotonated (nu-cleotide) and the other was protonated (polyamine), and the molecular complex formation was a result of an ion-ion or ion-dipole reaction [26, 32–35]. In the systems amino acid/nucleotide, the pH ranges of protonation of both ligands overlap and each ligand has both positive and negative reaction centres. The formation of a molecular complex can be described by the equation: H_*x*_Asp + H_*y*_(CMP) ⇆ (Asp)_H(*x*+*y*−*n*)_ (CMP) + *n*H^+^. The release of a proton in this reaction permits the use of the potentiometric method for determination of the composition and stability constants of the adducts. Analogous procedures were used for the investigation of the coordination compounds. The modes of interactions were determined on the basis of the spectroscopic measurements in the pH ranges in which particular complexes dominate, as established on the basis of the equilibrium study.

### 3.1. Asp/nucleotide metal-free systems

Table 1 presents the composition, overall stability constants (log *β*), and the equilibrium constants of the formation (log *K*_*e*_) of molecular complexes appearing in Asp/CMP systems, determined from the computer analysis of the potentiometric titration data.

**Figure 1:**
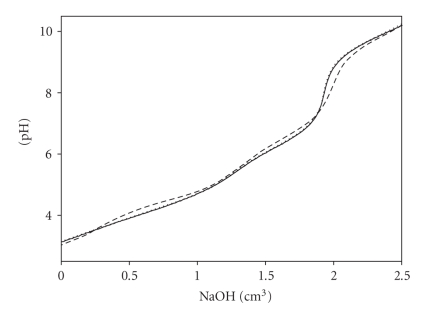
Experimental and simulated titration curves for the Asp/CMP system: dotted line: experimental curve; solid line: simulated curve (an adduct formation was taken into account); dashed line: simulated curve (an adduct formation was not taken into account).

As it was described earlier [28, 29, 36], the occurrence of noncovalent interactions between the ligands and the formation of molecular complexes in the system studied is indicated by the coincidence of the titration curves obtained experimentally and those obtained by computer simulation (with the use of the determined *β* values) as given in Figure 1.

Deprotonation of the phosphate group of the nucleotide begins at low pH (log *K*_3_ ∼ 0.4, [37, 38]), beyond the range of the study. Subsequent stages of deprotonation correspond to the abstraction of a proton from the endocyclic nitrogen atom N(3) from CMP and of another proton from the phosphate group. The deprotonation of the aspartic acid molecule begins with the proton abstraction from the carboxyl group C_(1)_, followed by dissociation of the –C_(4)_OOH and the –NH_3_^+^ group [39, 40].

**Figure 2:**
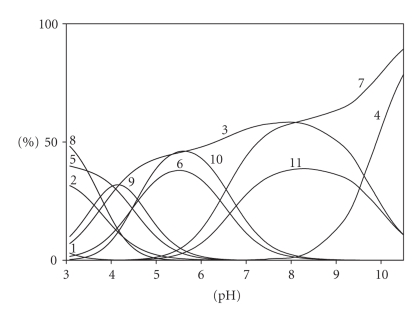
Distribution diagram for the Asp/CMP system; percentage of the species refers to total ligands. (1) H_3_Asp; (2) H_2_Asp; (3) HAsp; (4) Asp; (5) H_2_CMP; (6) HCMP; (7) CMP; (8) (Asp)H_4_(CMP); (9) (Asp)H_3_(CMP); (10) (Asp)H_2_(CMP); (11) (Asp)H(CMP); C_Asp_ = 6 × 10^−3^ M; C_CMP_ = 6 × 10^−3^ M.

Because of the different stoichiometric compositions of particular species, the overall stability constants log *β* cannot be directly applied in analysis of the character of interactions. Therefore, the efficiency of bonding was estimated on the basis of the equilibrium constants calculated, for example, for the species (Asp)H_2_(CMP): log *K*_*e*_ = log *β*_(Asp)H_2_(CMP)_ − log *β*_(HAsp)_ − log *β*_H(CMP)_. On the basis of the protonation constants of the ligands (Table 1) and the pH ranges of occurrence of individual species (Figure 2), the substrates in the adduct formation reactions were identified.

The complex (Asp)H_4_(CMP) appears at pH below 4.0 (Figure 2) in the range in which one of the carboxyl groups of Asp and partly the –PO_4_^2−^ group from CMP are deprotonated [41–44]. In the ^13^C NMR spectra, the chemical shifts of the signals assigned to C(2) and C(4) from the vicinity of N(3) of the nucleotide changed by 0.982 and 0.760 ppm (pH 3.0), respectively, (Table 2) indicate that the protonated N(3)H from CMP is a positive centre of weak interactions.

The lack of significant changes in the ^31^P NMR spectrum suggests that the partly protonated (however negative) phosphate group is not active, which is a result of the repulsion from the negative carboxyl group present in the neighbourhood of –NH_3_^+^ group of Asp. The change in the chemical shift of the signal assigned to C_(1)_ by 0.049 ppm suggests that the negative centre of interaction is the deprotonated carboxyl group from Asp. (As established earlier, the energy of the noncovalent interactions does not correspond directly with the chemical shift value [27, 29, 34]). The protonated carboxyl group is not involved in the interactions as evidenced by the lack of changes in the chemical shifts of the NMR signals assigned to the carbon C_(4)_ atom. In the (Asp)H_3_(CMP) adduct, dominant at a pH close to 4, the N(3)H group of CMP remains a positive centre of interactions as indicated by the changes in the NMR chemical shifts, while the group –C_(4)_OO^−^ becomes a negative centre of interaction (Table 2), which corresponds to the deprotonation of the second carboxyl group. The phosphate group remains inactive. The involvement of only one centre from each ligand in the interactions is confirmed by similar values of the equilibrium constants of the tri- and tetraprotonated complexes of log *K*_*e*_ = 2.84 and 2.87, respectively.

With increasing pH, the deprotonation of the N(3)H group from the pyrimidine ring of CMP takes place. The (Asp)H_2_(CMP) adduct starts forming from a pH close to 4 and reaches its maximum concentration at a pH of about 5.5. The changes in the chemical shifts of the carbon atoms C(2) and C(4), neighbouring the N(3) atom of the nucleotide at pH 5.5, in the region of domination of the (Asp)H_2_(CMP) adduct, are 0.440 and 0.593 ppm. This observation indicates that N(3) is a centre of interactions, however, since it is already deprotonated, an inversion of interactions takes place and the N(3) atom becomes a negative centre of interaction. The ^31^P NMR spectrum of (Asp)H_2_(CMP) at pH 5.5 does not show any significant changes in the chemical shift of the phosphorus atom (Table 2), which indicates a low efficiency of the phosphate group.

**Figure 3:**
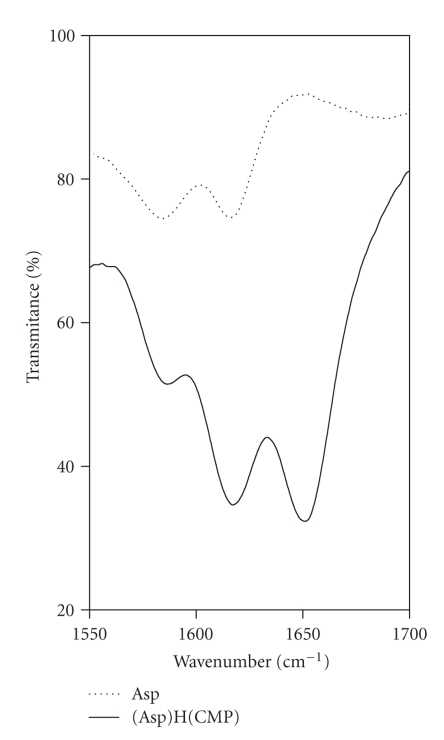
IR spectra of Asp and (Asp)H(CMP) adduct pH = 8; C_Asp_ = 0.2 M, C_CMP_ = 0.2 M.

Above the physiological pH, the formation of (Asp)H(CMP) begins and it dominates at a pH of about 8. In these complexes, the phosphate group from the nucleotide is engaged in noncovalent interactions with amino acid as proved by the changes in the position of the signal of ^31^P NMR, by 0.105 ppm at pH = 8. Changes in the chemical shifts of the C(2) and C(4) atoms from CMP by 0.191 and 0.198 ppm, respectively, indicate that the deprotonated N(3) atom is another negative centre of interaction. On the other hand, as follows from the changes in the chemical shift of the signal assigned to C_(2)_ (1.048 ppm), the protonated amine group from Asp (positive, log *K*_1_ = 9.6) takes part in the interaction. In the molecule of the nucleotide, there are no positive reaction centres, thus the intermolecular interactions of –COO^−^ are impossible, (similar to the intramolecular interactions). Because of the lack of another active centre in the Asp molecule, no increase in the log *K*_*e*_ value of the monoprotonated complex, relative to that of diprotonated one, is observed. The unexpected changes in the chemical shift of the signals assigned to C_(1)_ and C_(4)_ from Asp in the ^13^C NMR spectrum (Table 2) are a consequence of the interaction of the carboxyl groups (hard base) with Na^+^ ions (hard acids) present in the systems studied. In the additional ^13^C NMR spectrum taken for the (Asp)H(CMP) adduct in the system containing (C_2_H_5_)_4_NOH instead of NaOH, the changes were insignificant (at pH 8: 0.007 and 0.020 ppm for C_(1)_ and C_(4)_, resp.). The positions of the signals assigned to C_(1)_ and C_(4)_ from Asp in the spectra of the Na^+^ free system Asp/CMP were compared to those in the spectra of Asp, without sodium ions. (This results also explains the unexpected shifts in the signals assigned to carbon atoms from carboxylate groups in some other studied systems.) The above conclusions are confirmed by analysis of IR spectra recorded in the same conditions as NMR ones. A comparison of the position of the IR band assigned to –COO^−^ of the amino acid (1615 and 1584 cm^−1^) and the (Asp)H(CMP) adduct (1617 and 1586 cm^−1^) shows that these groups are not involved in the ligand-ligand interactions (Figure 3), because no changes in the band positions were observed.

**Scheme 2:**
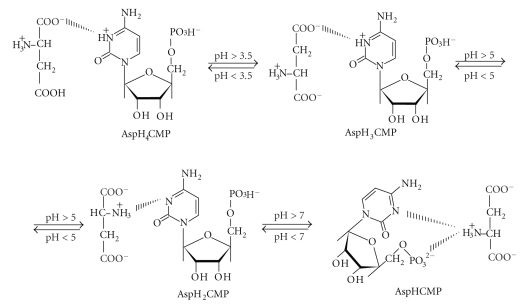
Noncovalent interaction in the Asp/CMP system.

In all adducts studied above, noncovalent interaction occurs with the inversion of interaction sites at a pH close to 3.5 and close to 7, as illustrated in Scheme 2. The pH values of the inversion correspond to the values of the protonation constants, and changes in the mode of interaction are a result of the deprotonation of the second carboxyl group of Asp, endocyclic N(3)H, and the phosphate group of CMP. No significant changes are noted in the acid-base equilibria of the ligands as is usually the case when metal-ligand bonds are formed, which confirms that the interactions are weak, noncovalent.

### 3.2. Cu/Asp binary systems

The stability constants of Cu(II) complexes with Asp were determined in the same conditions in which the heteroligand complexes in the ternary systems are formed (Table 3). The results are in agreement with the earlier reported data [39, 40, 45, 46].

In the pH range from 2.5 to 6, a protonated species CuH(Asp) is formed, while at pH 5.5, the dominant complex is Cu(Asp), binding about 80% of copper ions. The species Cu(Asp)_2_ dominates at a pH from 7 to 10, while the hydroxocomplex Cu(Asp)(OH) begins forming from a pH close to 6 reaching a maximum concentration above pH 10.5.

The UV-Vis and EPR spectral parameters (Table 4) at pH 3, at which the CuH(Asp) complex dominates: *λ*_max_ = 756 nm, *g*_∥_ = 2.413 and *A*_∥_ = 134, indicate that the oxygen atoms from the Asp carboxyl groups are exclusively involved in the coordination of the copper ions [26, 32, 47, 48], while the protonated amine group –NH_3_^+^ is blocked to coordination and does not take part in the interactions ({O_x_} chromophore). Conclusions concerning the mode of coordination were drawn on the basis of an analysis of the relation between the spectral parameters and the number of donor atoms in the inner sphere of Cu(II) coordination. For tetragonal and square pyramidal species, the ground state is normally d_*x*2−*y*2_ or rarely d_*xy*_. As earlier established for Cu‐N_*x*_ (*x* = 1–6) and Cu‐N_*x*_O_*y*_ (*x* = 0–4, *y* = 0–6) in planar bonding, the value of *g*_∥_ decreases and that of *A*_∥_ increases [47, 49]. In general, the value of *g*_∥_ is changed in the order CuO_4_ > CuO_2_N_2_ > CuON_3_ > CuN_4_. Weak bonding of the donor atoms at the axial position is a result of the interactions between the 4s and 4p metal orbitals and the ligand orbitals. The hyperfine coupling constant was experimentally observed to decrease with increasing electron density of the 4s orbital [47].

The carboxyl group involvement is confirmed by the changes in the ^13^C NMR chemical shifts of the carbon atoms from the two carboxyl groups: C_(1)_ and C_(4)_ by 1.536 and 1.163 ppm, respectively (Table 5).

The increase in the equilibrium constant of the formation of Cu(Asp) by approximately 5.5 log *K*_*e*_ unit relative to the value for the protonated complex (Table 3) points to the involvement of the deprotonated amine group –NH_2_ (for pH above 5) in the coordination of Cu(Asp) species, which is consistent with the model proposed by Chaberek and Martell, later confirmed in [39, 50]. Moreover, in the electronic absorption spectrum, the *λ*_max_ is shifted toward higher energy—from 756 to 700 nm, which points to the engagement of the nitrogen atom from Asp in metal coordination, (besides the oxygen atoms). The {N,Ox} type coordination was also confirmed by the EPR results as at pH = 5, *g*_∥_ = 2.301 and *A*_∥_ = 171. The attachment of a subsequent molecule of amino acid to the anchoring Cu(Asp), according to the equation Cu(Asp) + Asp ⇆ Cu(Asp)_2_, leads to a decrease in the equilibrium constant by about 2 log *K*_*e*_, units, so by a value suggesting the same mode of coordination in the 1:1 and 1:2 complexes, taking into consideration the statistical relations. The spectral parameters obtained from the UV-Vis and EPR spectra for the Cu(Asp)_2_ complex are *λ*_max_ = 630 nm, *g*_∥_ = 2.257 nm, and *A*_∥_ = 187 (Table 4) indicating (in agreement with Gampp et al. [48]) the formation of the {N2,Ox} chromophore with the participation of the deprotonated nitrogen and oxygen atoms from the carboxyl groups of the amino acid, which also follows from the changes in the ^13^C NMR spectra. At pH 8, at which the Cu(Asp)_2_ species reaches a maximum concentration, the changes in chemical shifts of the carbon atoms C_(1)_, C_(2)_, and C_(4)_ are 0.595, 0.602, and 0.755 nm, respectively.

The Cu(Asp)(OH) complex occurs in the same pH range as that of the dominant Cu(Asp)_2_ species and, therefore, no spectra could be taken.

### 3.3. Cu(II)/Asp/CMP system

**Figure 4:**
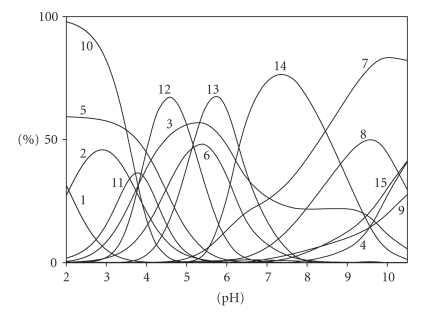
Distribution diagram for the Cu(II)/Asp/CMP system; percentage of the species refers to total metal. (1) H_3_Asp; (2) H_2_Asp; (3) HAsp; (4) Asp; (5) H_2_(CMP); (6) H(CMP); (7) CMP; (8) Cu(Asp)_2_; (9) Cu(Asp)(OH); (10) Cu(Asp)H_4_(CMP); (11) Cu(Asp)H_3_(CMP); (12) Cu(Asp)H_2_(CMP); (13) Cu(Asp)H(CMP); (14) Cu(Asp)_2_H(CMP); (15) Cu(Asp)(CMP)(OH); C_Cu^2+^_ = 1 × 10^−3^ M; C_Asp_ = 2.5 × 10^−3^ M; C_CMP_ = 2.5 × 10^−3^ M.

Analysis of the equilibria in the ternary systems was performed using the protonation constants and overall stability constants (log *β*) of the complexes forming in the binary systems of Cu(II)/CMP [20]. In the system Cu(II)/Asp/CMP, the following complexes were found: the protonated ones Cu(Asp)H_4_(CMP), Cu(Asp)H_3_(CMP), Cu(Asp)H_2_(CMP), Cu(Asp)_2_H(CMP), and the hydroxocomplex Cu(Asp)(CMP)(OH). Table 3 presents the results of a computer analysis of titration curves in ternary systems with Cu(II) ions, Asp, and CMP. The Cu(Asp)H_4_(CMP) species was already observed to form at a pH below 2, binding over 80% of Cu(II) ions at pH 3 (Figure 4). Taking into regard the number of hydrogen atoms in the species studied, the position of the d-d bands, and the EPR parameters (at pH 3, *λ*_max_ = 780 nm, *g*_∥_ = 2.409, and *A*_∥_ = 138, Table 4), one can conclude that the Cu(II) ion coordination occurs only by oxygen atoms from Asp.

The composition (in particular the number of hydrogen atoms blocking the coordination) of the Cu(Asp)H_4_(CMP) complex and the pH range of its occurrence suggest that it is a molecular complex. Conclusions concerning the mode of interactions could be achieved on the basis of spectroscopic studies. The UV-Vis and EPR data (*λ*_max_ = 780 nm, *g*_∥_ = 2.409, and *A*_∥_ = 138, Table 4) indicate bonding of copper(II) ion only via one oxygen atom (coordination Cu-O in an inner sphere) with the involvement in metallation of a deprotonated carboxyl group C_(1)_ from the amino acid (the second carboxyl group is protonated and blocked for coordination). On the basis of this essential finding, the next stages of the analysis were performed. In the ^13^C NMR spectrum of this species (pH = 3), the change in the chemical shift of C_(1)_ from Asp is 1.371 ppm. Moreover, the changes in the signals assigned to the Asp carbon atoms C_(2)_ (0.460 ppm) and C_(4)_ (1.094 ppm) and originated from the nucleotide carbon atoms C(2) and C(4) by 0.463 and 0.528 ppm, respectively, and the change in the ^31^P NMR of CMP by 0.166 ppm confirm clearly the hypothesis relating to the intermolecular interactions between the protonated complex of Cu(II) with Asp and the protonated CMP molecule located in the outer coordination sphere. The noncovalent interaction centre becomes the protonated Asp amine group, the CMP phosphate group, and N(3)H.

With increasing pH and deprotonation of the carboxyl group C_(4)_ in the Asp molecule, the Cu(Asp)H_3_(CMP) species is formed. The protonated nucleotide with the donor atoms blocked is probably involved in noncovalent interactions with the anchoring CuH(Asp) species. The pH range of formation of this species overlaps the ranges of formation of other ones (Figure 4), which makes it impossible to perform spectral studies and to explain their mode of coordination.

The Cu(Asp)H_2_(CMP) complex begins to form from a pH close to 3 in parallel with the deprotonation of the nitrogen atom N(3) of the nucleotide, and at pH 4.5, it binds about 70% of Cu(II) ions. The position of the absorption band in the UV-Vis spectrum in the pH range of the complex domination *λ*_max_ = 710 nm and the values of the EPR parameters *g*_∥_ = 2.275 and *A*_∥_ = 179 (Table 4) [48, 51] indicate formation of the {N,Ox} type chromophore and it provides the basis of information concerning the character of interaction. In the NMR spectrum of Cu(Asp)H_2_(CMP), the changes in the chemical shifts of the carbon atoms C_(1)_ and C_(4)_ from Asp (0.069 and 0.080 ppm, resp.) and the carbon atoms C(2) and C(4) from the nucleotide (1.042 and 0.932 ppm, resp.), together with the changes in the ^31^P NMR signal (0.080 ppm), point to the involvement in metallation of the deprotonated N(3) atom, the phosphate group of CMP, and the oxygen atoms from the carboxyl group of Asp. As follows from the value of log *K*_*e*_ = 6.08 for Cu(Asp)H_2_(CMP) (so for the reaction of HCMP attachment to the anchoring CuHAsp), much higher than that for Cu(CMP) formation log *K*_*e*_ = 2.71 [20], there is a noncovalent intramolecular ligand-ligand interaction in the Cu(Asp)H_2_(CMP) complex that additionally stabilises it (the presence of weak interactions is confirmed by changes in the chemical shift of C_(2)_ from Asp (0.643 ppm) located in the proximity of the NH_3_^+^ group). The equilibrium constant of the Cu(Asp)H_2_(CMP) complex formation is by about 3 orders of magnitude higher than that of the adduct (Asp)H_2_(CMP), which is a result of significant differences in the character of the bond (in the metal-free system the bond is weak, noncovalent, but in the copper complex both ligands bind the metal ions with additional intramolecular interaction).

**Scheme 3:**
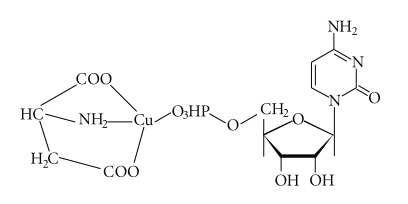
Tentative mode of interaction in the Cu(Asp)H(CMP) complex.

The Cu(Asp)H(CMP) complex begins to form from a pH close to 4, and at pH = 6, it binds 80% of the Cu(II) ions. In the pH range of its domination *λ*_max_ = 700 nm, the EPR parameters are *g*_∥_ = 2.269 and *A*_∥_ = 182, which implies the involvement of one donor nitrogen atom and oxygen atoms from the ligand molecules in the coordination ({N,Ox} chromophore). The NMR spectra reveal changes in the positions of signals coming from the carbon atoms of the Asp carboxyl groups (C_(1)_ 0.769 ppm and C_(4)_ 0.809 ppm), the phosphorus atom of CMP (^31^P NMR 0.190 ppm), and the carbon atom located close to the Asp amine group (C_(2)_ 0.782 ppm). Moreover, changes in positions of the signal originated from the CMP carbon atoms located in the proximity of N(3) (C(2), 0.486 ppm and C(4), 0.772 ppm) are observed. The question of which nitrogen atoms (either from the Asp amine group or the endocyclic N(3) atom) from CMP are involved in the metallation has been solved by comparison of the thermodynamic stability of the two possible species. The first species corresponds to the formation of a stable system coupled with five- and six-membered rings (Scheme 3), while the second corresponds to the formation of an unstable seven-membered ring and a macrochelate structure with a coordination via the –PO_4_^2−^ group and N(3) atom from CMP.

In the pH range 7–8, the dominant species is Cu(Asp)_2_H(CMP). The positions of the d-d bands and the EPR parameters (pH = 8, Table 4) imply the formation of an {N2,Ox} type chromophore. The Cu(Asp)H(CMP) molecule accepts another amino acid molecule. As follows from the changes in NMR signals assigned to the carbon atoms of the carboxyl group (C_(1)_ 0.809 ppm and C_(4)_ 0.689 ppm) and from those in the proximity of the Asp amine group (C_(2)_ 0.749 ppm) as well as the changes in the chemical shifts assigned to the C(2) and C(4) atoms from the neighbourhood of N(3) in CMP by 0.860 and 0.858 ppm, respectively, and changes in the chemical shifts in the ^31^P NMR spectrum of the nucleotide (0.129 ppm), the coordination involves the oxygen atoms of the three carboxyl groups and the amine group from the Asp molecule and the endocyclic N(3) atom together with the phosphate group from CMP (monofunctional character of the second Asp ligand). The hydroxocomplex Cu(Asp)(CMP)(OH) begins to form from a pH close to 8, and at pH = 10.5, this species dominates (the equilibrium Cu(Asp)_2_H(CMP) + OH^−^ ⇆ Cu(Asp)(CMP)(OH) + H(Asp). The maximum of the band in the UV-Vis spectrum is at 630 nm, and the EPR parameters are *g*_∥_ = 2.258 and *A*_∥_ = 183, which corresponds to the {N2,Ox} chromophore with coordination via the nitrogen atom N(3), the phosphate group from CMP, the oxygen atom from the Asp amine group, oxygen atoms from the carboxyl groups of the amino acid, and oxygen atoms from the OH group.

## 4. CONCLUSIONS

The main reaction centres in complexes formed as a result of noncovalent interactions in metal-free systems at low pH are the nitrogen N(3)H group from CMP and carboxyl groups from Asp. With increasing pH, the efficiency of the interactions of the Asp carboxyl group decreases, while that of the amine group increases. For a pH up to about 5, the protonated N(3)H from the nucleotide is the positive centre of weak interactions and at a higher pH, an inversion occurs and it becomes a negative centre. Above a pH of about 7, the involvement of the phosphate group occurs in the interaction. The change in the mode of interactions corresponds to the pH ranges of protonation of particular ligands. No significant acid-base shifts are observed in the systems studied, which confirms the hypothesis that the interactions are weak. Introduction of the metal ion into the binary system changes the character of the reaction in the ternary system relative to that in the metal-free systems. Particularly important is a significant increase in the efficiency of the phosphate group from CMP in the noncovalent interactions in the Cu(Asp)H_4_(CMP) molecular complex. In the (Asp)H_4_(CMP) adduct which forms in the same pH range, the –PO_4_H^−^ group is ineffective.

In the ternary systems, Cu(II)/Asp/CMP coordination is realised through the carboxyl groups from Asp, and starting from a pH close to 4, also the phosphate groups from CMP and the endocyclic N(3) atom are involved in interactions. At low pH, the carboxyl groups from the amino acid are the main metal binding site, but with increasing pH, their efficiency decreases to the advantage of the phosphate groups, as follows from the analysis of the log *K*_*e*_ values. The presence of metal ions changes the character of noncovalent interactions, whereas the introduction of an additional ligand CMP into the binary system Cu(II)/Asp changes the mode of coordination. For instance, in the Cu(Asp) complex, the coordination is realised via the oxygen atoms from the carboxyl groups and the amine group (five-membered ring), while in the ternary complex Cu(Asp)H(CMP) occuring in the same pH range, the involvement of the phosphate group from CMP in metal binding leads to a change in the mode of coordination and the formation of a structure with a system of coupled five- and six-membered rings.

## Figures and Tables

**Table 1:** Overall stability constants (log *β*) and equilibrium constants (log *K*_*e*_) of adducts formation in Asp/CMP system.

Species	log *β*	log *K*_*e*_
H_3_Asp	15.42 (3)	2.06
H_2_Asp	13.36 (1)	3.73
HAsp	9.63 (1)	9.63

H_2_CMP	10.90 (1) [20]	4.47
HCMP	6.42 (2) [20]	6.42

(Asp)H_4_(CMP)	27.13 (4)	2.87
(Asp)H_3_(CMP)	23.37 (3)	2.84
(Asp)H_2_(CMP)	18.79 (3)	2.74
(Asp)H(CMP)	12.01 (3)	2.38

**Table 2:** Differences between ^13^C NMR and ^31^P NMR chemical shifts for the ligands in the Asp/CMP system in relation to the free ligands [ppm].

	Asp				CMP					
pH	C_(1)_	C_(2)_	C_(3)_	C_(4)_	C(2)	C(4)	C(5)	C(6)	C(5′)	P
3.0	0.049	0.025	0.022	0.002	0.982	0.760	0.057	0.048	0.044	0.018
4.2	0.000	0.013	0.070	0.054	0.861	0.661	0.040	0.047	0.060	0.025
5.5	0.053	0.047	0.027	0.040	0.440	0.593	0.068	0.054	0.099	0.040
8.0	0.454	1.048	0.227	1.322	0.191	0.198	0.068	0.064	0.111	0.105

**Table 3:** Overall stability constants (log *β*) and equilibrium constants (log *K*_*e*_) for the complexes of Cu(II) ions with Asp or CMP and Cu(II) ions with Asp and CMP.

Equilibrium	log *β*	log *K*_*e*_
Cu + CMP ⇆ Cu(CMP)	2.71 (7) [20]	2.71
Cu + CMP + H_2_O ⇆ Cu(CMP)(OH) + H^+^	−4.26 (6) [20]	

Cu + H^+^ + Asp ⇆ CuH(Asp)	12.78 (5)	3.15
Cu + Asp ⇆ Cu(Asp)	8.76 (3)	8.76
Cu + 2Asp ⇆ Cu(Asp)_2_	15.35 (4)	6.59
Cu + Asp + H_2_O ⇆ Cu(Asp)(OH) + H^+^	0.79 (9)	

Cu + 4H^+^ + Asp + CMP ⇆ Cu(Asp)H_4_(CMP)	32.86 (5)	
Cu + 3H^+^ + Asp+CMP ⇆ Cu(Asp)H_3_(CMP)	29.14 (5)	5.46
Cu + 2H^+^ + Asp + CMP ⇆ Cu(Asp)H_2_(CMP)	25.28 (4)	6.08
Cu + H^+^ + Asp + CMP ⇆ Cu(Asp)H(CMP)	20.12 (4)	4.94
Cu + H^+^+ 2Asp + CMP ⇆ Cu(Asp)_2_H(CMP)	29.87 (4)	
Cu + Asp + CMP + H_2_O ⇆ Cu(Asp)(CMP)(OH) + H^+^	4.69 (4)	

**Table 4:** Vis and EPR spectral data for Cu(II)/Asp and Cu(II)/Asp/CMP systems.

Species	pH	*λ*_max_ [nm]	*ε* [M^−1^cm^−1^]	EPR	
				*g*_∥_ = (dm^3^/mol·cm^3^)	*A*_∥_(10^−4^cm^−1^)
CuH(Asp)	3	756	27	2.413	134
Cu(Asp)	5	700	40	2.301	171
Cu(Asp)_2_	8	630	76	2.257	187

Cu(Asp)H_4_(CMP)	3	780	25	2.409	138
Cu(Asp)H_2_(CMP)	4.5	710	29	2.275	179
Cu(Asp)H(CMP)	6	700	30	2.269	182
Cu(Asp)_2_H(CMP)	8	660	49	2.261	182
Cu(Asp)(CMP)(OH)	10.5	630	96	2.258	183

**Table 5:** Differences between ^13^C NMR and ^31^P NMR chemical shifts for the ligands in the Cu(II)/Asp and Cu(II)/Asp/CMP systems in relation to metal-free systems [ppm].

Systems	Asp					CMP					
	pH	C_(1)_	C_(2)_	C_(3)_	C_(4)_	C(2)	C(4)	C(5)	C(6)	C(5′)	P
Cu(II)/Asp	3.0	1.536	0.129	0.122	1.163	—	—	—	—	—	—
	5.5	0.050	0.070	0.020	0.050	—	—	—	—	—	—
	8.0	0.595	0.602	0.169	0.755	—	—	—	—	—	—

Cu(II)/Asp/CMP	3.0	1.371	0.460	0.147	1.094	0.463	0.528	0.075	0.085	0.057	0.166
	4.5	0.069	0.643	0.113	0.080	1.042	0.932	0.052	0.047	0.051	0.080
	6.0	0.769	0.782	0.142	0.809	0.486	0.772	0.062	0.046	0.076	0.190
	8.0	0.809	0.749	0.152	0.689	0.860	0.858	0.071	0.064	0.062	0.129
